# L Band Brightness Temperature Observations over a Corn Canopy during the Entire Growth Cycle

**DOI:** 10.3390/s100706980

**Published:** 2010-07-20

**Authors:** Alicia T. Joseph, Rogier van der Velde, Peggy E. O’Neill, Bhaskar J. Choudhury, Roger H. Lang, Edward J. Kim, Timothy Gish

**Affiliations:** 1 Hydrological Sciences Branch/614.3, Hydrospheric and Biospheric Sciences Laboratory, NASA/Goddard Space Flight Center, Greenbelt, MD 20771, USA; E-Mails: Peggy.E.ONeill@nasa.gov (P.E.O.); Bhaskar.J.Choudhury@nasa.gov (B.J.C.); Edward.J.Kim@nasa.gov (E.J.K.); 2 International Institute for Geo-Information Science and Earth Observation (ITC), Hengelsostraat 99, P.O. Box 6, 7500 AA Enschede, The Netherlands; E-Mail: velde@itc.nl; 3 Department of Electrical Engineering & Computer Sciences, the George Washington University, Washington, DC 20052, USA; E-Mail: lang@gwu.edu; 4 USDA-ARS Hydrology and Remote Sensing Laboratory, Building 007, BARC-WEST, Beltsville, MD 20705, USA; E-Mail: Timothy.Gish@ars.usda.gov

**Keywords:** field campaign, L-band radiometry, vegetation effects, surface roughness

## Abstract

During a field campaign covering the 2002 corn growing season, a dual polarized tower mounted L-band (1.4 GHz) radiometer (LRAD) provided brightness temperature (*T*_B_) measurements at preset intervals, incidence and azimuth angles. These radiometer measurements were supported by an extensive characterization of land surface variables including soil moisture, soil temperature, vegetation biomass, and surface roughness. In the period May 22 to August 30, ten days of radiometer and ground measurements are available for a corn canopy with a vegetation water content (*W*) range of 0.0 to 4.3 kg m^−2^. Using this data set, the effects of corn vegetation on surface emissions are investigated by means of a semi-empirical radiative transfer model. Additionally, the impact of roughness on the surface emission is quantified using *T*_B_ measurements over bare soil conditions. Subsequently, the estimated roughness parameters, ground measurements and horizontally (H)-polarized *T*_B_ are employed to invert the H-polarized transmissivity (*γ*_h_) for the monitored corn growing season.

## 1. Introduction

Low frequency passive microwave observations have been intensively studied for their potential of retrieving soil moisture e.g., [1–3]. Studies have demonstrated that when an appropriate characterization of vegetation, soil surface roughness and dielectric properties are applied, soil moisture can be retrieved fairly accurately from the brightness temperatures (*T*_B_’s) measured by microwave radiometers e.g., [4,5]. As a result, the Soil Moisture and Ocean Salinity (SMOS [6]) mission is the first of three L-band radiometers designed for global soil moisture monitoring purposes to be launched. In the near future, the Aquarius and Soil Moisture Active Passive (SMAP [7]) missions will follow; their expected launch dates are in spring 2010 and in 2015, respectively. With this increased availability of low frequency spaceborne radiometer observations, new opportunities arise for monitoring soil moisture globally.

Among the challenges in retrieving soil moisture from *T*_B_ measurements is to account for soil surface roughness and vegetation effects. Most retrieval approaches utilize similar radiative transfer equations [8–10], in which the effects of vegetation are parameterized by the vegetation transmissivity (*γ*) and the single scattering albedo (*ω*). Pardé *et al*. [11] concluded that for retrieving soil moisture globally the *ω* can be assumed constant over time. Conversely, the *γ* changes over time because its magnitude is proportional to the biomass and is also affected by vegetation geometry e.g., [12,13]. Moreover, the *γ* is known to depend on the sensing configuration (e.g., frequency, view angle and polarization) e.g., [14–16].

Methods for estimating the *γ* use either multiple channel microwave data or ancillary data. A direct estimation of the *γ* from microwave data is preferred because the ancillary data needed at a global scale for soil moisture retrieval may not be available. However, its dependence on the instrument parameters complicates the inversion of *γ* from *T*_B_’s measured at different frequencies, view angles and polarizations. Large scale soil moisture monitoring studies e.g., [17–19] have, therefore, frequently adopted the ancillary data approach to determine the *γ*, which has been extensively described in the scientific literature e.g., [20,21]. For this characterization, the *γ* is related to the vegetation optical depth (*τ*), which is estimated as a function of the vegetation water content (*W*) and a crop-specific empirical parameter, *b*, which depends on the instrument parameters.

Various implementations of this approach within soil moisture retrieval algorithms have been reported. For example, Jackson *et al*. [8] used a land cover map to define for each crop type a specific *b* value and utilized the Normalized Difference Vegetation Index (NDVI) to estimate the *W*. Similarly, Bindlish *et al*. [22] adopted the NDVI as a proxy for the *W*, but inverted the *b* values from dual-polarized X-band (10.65 GHz) *T*_B_ by assuming that the *τ* is the same for both horizontally (H) and vertically (V) polarized data. This polarization dependence is taken into account by the SMOS level 2 processor [12] as its effect is expected to be more significant at the lower L-band frequency. In addition, a more sophisticated approach for modeling the view angle dependence of *τ* is included in SMOS processor because its *T*_B_’s are collected from different angles [12]. Further, apart from the NDVI also the Leaf Area Index (LAI) has been found to be a good estimator for *W* [23] and is used for the SMOS soil moisture retrieval.

Although iterative procedures for inverting the *τ* have been developed e.g., [11,22], initial values and uncertainty ranges for the *τ* are still needed as input. The selection of the appropriate parameterization for a specific land cover relies, however, often on parameter sets derived from *T*_B_ measurements collected during past intensive field campaigns e.g., [16,20]. By default, the validity of those parameterizations is restricted to the conditions for which they have been derived. Many of the past field campaigns covered, for example, a part of the growth cycle of agricultural crops. Therefore, the temporal evolution of the *γ* and *b* parameter throughout the growth cycle is not fully understood.

This paper contributes to this understanding by analyzing the L-band H-polarized *T*_B_’s measured throughout the complete 2002 corn (Zea mays L.) growth cycle. The utilized data set has been collected at one of the fields of the Beltsville Agricultural Research Center (BARC) by an automated tower mounted L-band (1.4 GHz) radiometer (called LRAD) starting from May 22nd till the beginning of September. These radiometer measurements were supported by a detailed land surface characterization, which took place about once every week and included measurements of the vegetation biomass, soil moisture and soil temperature. Despite mechanical difficulties with the scanning system of LRAD that produced gaps in the data record, a total of ten days distributed over the growing season of both radiometer and ground measurements are available covering a *W* range from 0.0 to 4.3 kg m^−2^.

The objective of this investigation is to evaluate the variations in the *γ* and the empirical parameter *b* over the monitored corn growth cycle. First, the impact of the surface roughness on the surface emission is quantified using the LRAD *T*_B_’s over bare soil conditions and an older data set collected at the BARC facility. Subsequently, the *γ* (and *b* parameter) are inverted from individual *T*_B_ measurements using the estimated roughness parameterization, and measured soil moisture and soil temperature. In addition, an analysis is presented of the sensitivity of the derived *b* parameters for uncertainties in the LRAD *T*_B_ and the assigned single scattering albedo (*ω*).

## 2. Theoretical Background

The starting point for the computation of microwave emission from vegetated surfaces is the semi-empirical radiative transfer approach by Mo *et al*. [24], which is based on the assumption that at L-band absorption is dominant over scattering,

(1) $$T_{B}^{p}=\left(1+R_{s}^{p}\gamma_{p}\right)\left(1-\gamma_{p}\right)\left(1\;-\omega_{p}\right)T_{v}+\left(1-R_{s}^{p}\right)\gamma_{p}T_{s}$$

where, $T_{B}^{p}$ is the polarized brightness temperature, $R_{s}^{p}$ is the soil surface reflectivity (= 1 − emissivity), *γ*_p_ is the transmissivity of vegetation, *ω*_p_ is the single scattering albedo, *T*_s_ and *T*_v_ are the soil and canopy temperatures, respectively, and superscript and subscript *p* indicates the polarization.

The first term on the right hand side of Equation (1) represents the microwave emission directly by vegetation and the radiation emitted by the vegetation reflected by the soil surface back towards the sensor. The second term quantifies the emission contribution from the soil, corrected for the attenuation by the vegetation layer.

The solution to the radiative transfer equation requires parameterization of the vegetation and soil surface layer radiative transfer properties. Further, temperatures of the vegetation and soil surface layer are required. However, when assuming the vegetation and soil surface are in thermal equilibrium with each other, *T*_s_ and *T*_v_ can be considered equal; this condition occurs typically near dawn. The required temperature is then considered representative for the emitting layer.

### 2.1. Emission from Soil

The surface emissivity is typically described in terms of the surface reflectivity. This is convenient because the microwave reflectivity under smooth surface conditions can be calculated using the Fresnel formulas for reflectivity (*R^p^*), which read for the H and V polarization,

(2) $$\begin{array}{ccc} R^{H}(\theta )={\left|\frac{\text{cos}\;\theta -{\left(\varepsilon_{r}-{\text{sin}}^{2}\;\theta \right)}^{\frac{1}{2}}}{\text{cos}\;\theta +{\left(\varepsilon_{r}-{\text{sin}}^{2}\;\theta \right)}^{\frac{1}{2}}}\right|}^{2} & \text{and} & R^{V}(\theta )={\left|\frac{\varepsilon_{r}\;\text{cos}\;\theta -{\left(\varepsilon_{r}-{\text{sin}}^{2}\;\theta \right)}^{\frac{1}{2}}}{\varepsilon_{r}\;\text{cos}\;\theta +{\left(\varepsilon_{r}-{\text{sin}}^{2}\;\theta \right)}^{\frac{1}{2}}}\right|}^{2} \end{array}$$

where, *ε*_r_ is the dielectric constant of soil, *θ* is the incidence angle.

In this study, the approach described by Wang and Choudhury [25] has been adopted to account for the effect of surface roughness on the reflectivity. This approach involves two parameters, where one parameter has an attenuating effect on the surface reflectivity and the other accounts for the depolarizing effect of the surface roughness,

(3) $$\begin{array}{ccc} R_{s}^{p}(\theta )=[(1-Q)R^{p}+QR^{q}]\text{exp}\;(-h) & \text{with} & h=h_{0}\;G(\theta ) \end{array}$$

where, *h*_0_ is roughness parameter given by 4*k*^2^*σ*^2^ with *k* as the wavenumber (2π/*λ*) and *σ* as the root mean square (*rms*) height of the surface height variations, *Q* is a polarization mixing factor, *G*(*θ*) is a function accounting for the angular dependence of surface roughness effect on surface emission and superscript *q* represents the polarization orthogonal to polarization *p*, which can be either H or V.

Originally, Wang and Choudhury [25] took the function *G*(*θ*) equal to cos^2^θ. However, Wang *et al*. [26] have found that the dependence of cos^2^θ is much too strong and replaced it by *G*(*θ*) = 1.0 for best fitting their data. The latter is initially adopted here.

### 2.2. Vegetation Effects on Soil Surface Emission

Within the radiative transfer approach, vegetation effects are characterized by two parameters: transmissivity (*γ*) and single scattering albedo (*ω*). The *ω* is a measure for the fraction of attenuated radiation scattered from the canopy,

(4) $$\omega_{p}=\frac{\kappa_{s}^{p}}{\kappa_{s}^{p}+\kappa_{a}^{p}}$$

where, $\kappa_{s}^{p}$ and $\kappa_{a}^{p}$ are the scattering and absorption coefficients, respectively.

These scattering and absorption coefficients can be obtained through application of the discrete medium approach e.g., [27,28], in which individual components of the vegetation layer (leaves and stems) are represented by elliptical and/or cylindrical dielectric scatterers. In some cases, the *ω* is assumed to be negligible or a variable dependent on the growth stage, which can be determined from controlled experiments where all other variables (e.g., soil moisture, temperature of emitting layer, surface roughness and transmissivity) are measured.

The transmissivity describes the amount of soil emission passing through the vegetation layer and is an important variable for quantification of the effect of vegetation on microwave emission. The one-way transmissivity through the canopy layer is formulated as,

(5) $$\gamma_{p}=\text{exp}\;(\frac{-\tau_{p}}{\text{cos}\;\theta })$$

where, *τ*_p_ is the polarization dependent optical depth or canopy opacity, which can be calculated using,

(5) $$\tau_{p}=k_{\mathrm{ep}}H_{v}$$

with

(6) $$k_{\mathrm{ep}}=\frac{4\pi }{\lambda }n_{o}\text{Im}\langle f_{\mathrm{pp}}\rangle$$

where, *H*_v_ is the canopy height, *k*_ep_ is a polarization dependent extinction coefficient, *n*_o_ is the number of phytoelements per unit volume, *λ* is the wavelength and Im〈*f_pp_*〉 is the imaginary part of the polarization dependent scattering matrix of the phytoelements in the forward direction.

Several studies [15,16,20] have shown that *τ*_p_ can be related to the vegetation water content as,

(7) $$\tau_{p}=b_{p}\cdot W$$

where, *W* is the vegetation water content (kg m^−2^) and *b*_p_ is an empirical parameter varying with crop type, canopy structure, wavelength, and polarization.

Equation (7) requires information about the *W* and *b*_p_ parameters for different types of vegetation. This approach has been frequently used for soil moisture retrieval purposes e.g., [1,8,19] and has been proposed as part of the soil moisture retrieval algorithms for current and future microwave radiometers e.g., [29]. The SMOS level 2 soil moisture retrieval processor adopts a similar approach relating the *τ*_p_ to the leaf area index (LAI) instead of the *W* [30].

## 3. The OPE^3^ Experiment

### 3.1. Site Description

**Figure 1.**
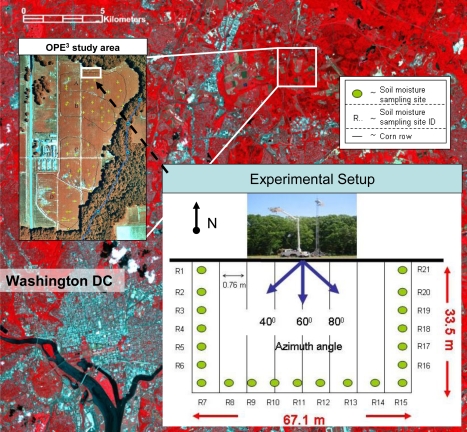
Location and schematization of the OPE^3^ remote sensing experimental setup in 2002.

The present study was conducted at Optimizing Production Inputs for Economic and Environmental Enhancement (OPE^3^) test site managed by the USDA-ARS (United States Department of Agriculture-Agricultural Research Service) [31]. The site consists of four adjacent watersheds with similar surface and sub-surface soil and water flow characteristics and covers an area of 25 ha near Beltsville, Maryland (Figure 1). Each of the four watersheds is formed from sandy fluvial deposits and has a varying slope ranging from 1% to 4%. The soil textural properties are classified as sandy loam with 23.5% silt, 60.3% sand, 16.1% clay, and bulk density of 1.25 g cm^−3^. A detailed description of the research activities can be found at http://hydrolab.arsusda.gov/ope3. (Verified December 23, 2009).

### 3.2. Ground Measurements

The *in-situ* measurement strategy was designed to provide ground information to supplement the radar and radiometer data acquisitions, and took place every Wednesday, rainy days excluded. In this paper, an analysis of the radiometer observations is presented. A description of the radar data set is given in Joseph *et al*. [32].

During the field campaign (May 10 to October 2, 2002) representative soil moisture, soil temperature, vegetation biomass (wet and dry) and surface roughness measurements were taken around the radiometer footprints. Soil moisture and soil temperature measurements were collected at twenty-one sites located at the edge of a 67.1 m × 33.5 m rectangular area depicted in Figure 1. Vegetation biomass and surface roughness measurements were taken around the study area at representative locations.

#### Soil moisture and soil temperature

Soil moisture was measured using gravimetric, portable impedance probe—Delta-T theta probe (The US Government does not endorse any specific brand of impedance probe for measuring soil moisture or any specific brand of digital thermometers), and buried impedance probe (Time Domain Reflectometry (TDR)) techniques. Soil samples of the top 6-cm soil layer were collected at the beginning of each day in conjunction with the theta probe measurements primarily for calibration purposes. Theta probe measurements were collected typically at 8:00, 10:00, 12:00 and 14:00 hours (USA Eastern). The buried TDR probes were installed at locations R5, R11 and R18 (Figure 1) at various depths (5, 10 and 20 cm) and insertion angles (horizontal, vertical, and 45 degrees).

**Figure 2.**
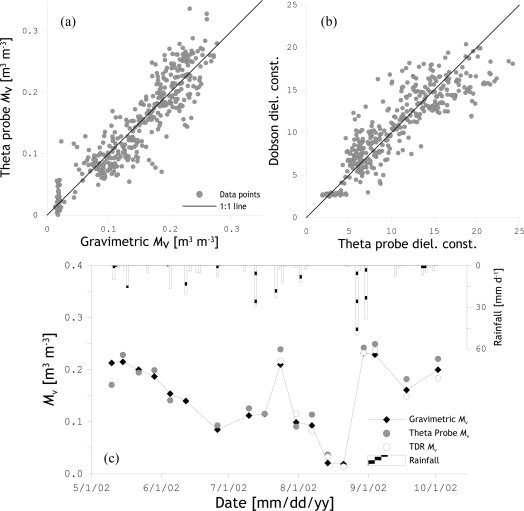
(a) Comparison of the calibrated theta probe against the gravimetric *M*_v_; (b) Comparison of the theta probe measured *ε*_r_ against the calculations made using the Dosbon soil mixing model; (c) *M*_v_ measured by the theta probe, TDR and gravimetric sampling technique plotted against time.

The soil dielectric constant (*ε*_r_) measured by the theta probe was converted to volumetric soil moisture (*M*_v_) values by fitting a linear regression function through the following relationship (Figure 2a),

(8) $$\sqrt{\varepsilon_{r}}=a_{0}+a_{1}\cdot M_{v}$$

where, *a*_0_ and *a*_1_ are regression parameters.

While general soil texture-specific parameters are available [33], a site specific calibration was performed. To achieve this, soil moisture determined gravimetrically from the soil samples was converted to *M*_v_ and used with concurrent probe observations to fit for each site a set of *a*_0_ and *a*_1_ values. Comparison of the calibrated theta probe *M*_v_ values with the gravimetric *M*_v_ (see Figure 2a) gives a root mean squared error (RSME) of 0.024 m^3^ m^−3^, which is comparable to calibration errors obtained with theta probe observations collected in several remote sensing campaigns [34]. In addition, Figure 2b shows the *ε*_r_ measured by the Theta probe plotted against the *ε*_r_ calculated with soil mixing model of Dobson *et al*. [35] using the soil texture and the gravimetric *M*_v_. The RMSE of 1.87 and coefficient of determination (R^2^) of 0.77 computed between the measured and calculated *ε*_r_ indicates that both methods for quantifying *ε*_r_ are in agreement with each other. Further, the *M*_v_ determined using the gravimetric, Theta probe and TDR probe techniques are displayed as time series in Figure 2c for comparison purposes. As shown by the plot, similar temporal soil moisture variations are observed by the three measurement approaches, which justify the use of each of their products.

Soil temperature measurements were taken manually at soil depths of 3- and 7-cm at each of the twenty-one sampling locations (annotated as R1 to R21 in Figure 1) throughout the experiment using Extech Instruments digital stem thermometers. On intensive sampling days the soil temperatures were measured at 8:00, 10:00, 12:00, 14:00 hours, and the measurements on other days were taken approximately every two days at 8:00 and 14:00 hours.

**Figure 3.**
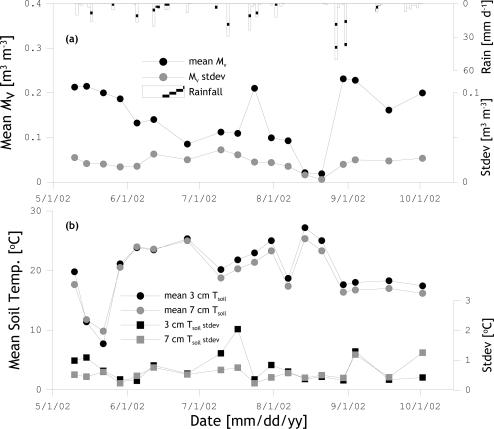
Mean and standard deviation of twenty-one soil moisture (a) and, 3-cm and 7-cm soil temperature; (b) measurements collected around the radiometer footprints.

Although the study area was selected to minimize the effects of land surface heterogeneity, small surface height and soil texture variations could potentially influence the representativeness of the measured soil moisture and temperature for the radiometer footprints. These effects are studied by presenting the temporal evolution of the mean and standard deviation (stdev) of the twenty soil moisture and soil temperature measurements in Figure 3. Figure 3a shows that the mean soil moisture changes in response to antecedent rain events. Also, the soil moisture stdev varies over time from 0.003 m^3^ m^−3^ under extreme dry conditions to 0.036 m^3^ m^−3^ in the mid soil moisture range. On average, however, the stdev remains quite stable around values of about 0.020 to 0.030 m^3^ m^−3^, which is compatible with the Theta probe calibration uncertainty of 0.024 m^3^ m^−3^. Further, the spatial temperature variability at soil depths of 3 cm (Figure 3b) and 7 cm (Figure 3c) is quite low with averaged stdev values of 0.73 and 0.58 °C, respectively. Given the fairly stable soil moisture stdev and low temperature stdev observed, the spatial heterogeneity around the footprint is expected to have only a minor effect on the representative mean of the twenty-one measurements for the radiometer footprint. The mean soil moisture and soil temperature values are, therefore, used for further analysis.

#### Vegetation

Corn was planted on April 17, reached peak biomass around July 24 and was harvested on October 2. Vegetation biomass and morphology were quantified through destructive measurements applied to 1 m^2^ area (approximately 12 plants) once every week at 8:00 am. The water content, fresh and dry biomasses were determined separately for the individual plant constituents, such as leaves stems and cobs (when present).

**Figure 4.**
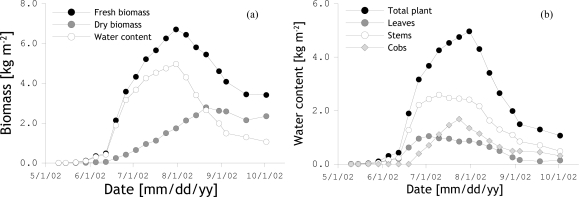
(a) Total plant water content, fresh and dry biomass plotted against time. (b) Water content in the leaves, stems and cobs plotted against time. The markers indicate the dates at which measurements were made.

Figure 4a shows the development biomasses and water content of the total plant over time and Figure 4b illustrates the temporal evolution of the water content in individual plant components. It follows from Figure 4b that in the beginning of the corn growing season, the canopy was primarily made up of leaves and stalks. In the middle of the growing season the stem contribution becomes more dominant and cobs’ water content increases to levels exceeding the leaf contribution. Near senescence, water content in the leaves is reduced further, whereas the contribution of the cobs to the total biomass remained constant.

#### Surface roughness

**Figure 5.**
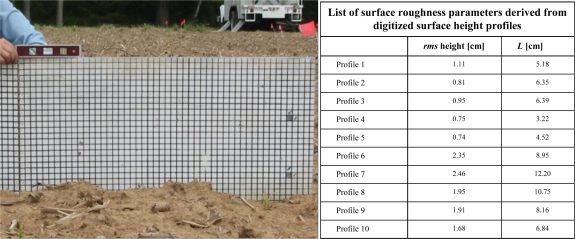
The left panel shows an example of a picture taken for surface roughness characterization and the right panel lists the derived surface roughness parameters.

During the experiment surface roughness was characterized on May 25 using the grid board technique. A 2-meter long grid board was placed in the soil and photographs were taken with the soil surface in front. In total, ten surface height profiles were recorded. The surface height profile in these pictures was digitized at a 0.5-cm interval, from which two roughness parameters were derived: the *rms* of the surface height and the correlation length (*L*). The averaged *rms* height and *L* for the ten observed surface roughness profiles were found to be 1.62 and 12.66 cm, respectively. Figure 5 shows an example of a photograph taken for this roughness characterization and lists the roughness parameters calculated from the digitized surface height profiles.

### 3.3. Radiometer

The deployed radiometer was a dual-polarized L-band passive microwave sensor, called LRAD. The instrument was mounted on a portable 18 m tower and was designed to collect data automatically (for this experiment every hour) at five incidence angles (25, 35, 45, 55, and 60 degrees) and three azimuth angles over a range of 40 degrees. LRAD had a 3 dB beam width of approximately 12 degrees, which corresponds to footprints varying from 4.5 to 15.5 meters for the 25 to 60 degrees incidence angle range. Mechanical difficulties with the scanning system restricted the LRAD data collection, and produced considerable gaps in the season-long record. Nevertheless, ten days of complete record (ground measurements and radiometer observations) were available for the present analysis.

Each LRAD data run consisted of a pre-calibration, a measuring sequence, and a post-calibration. During each of the two calibration periods one microwave observation was acquired from a microwave absorber target of known temperature (hot target) and one microwave observation was acquired of the sky (cold target), which has at L band an *T*_B_ of ∼5 K (3 K cosmic background radiation and 2 K atmospheric contribution). These two so-called “hot” and “sky” target observations can be used to calibrate, through linear interpolation, the radiometer observations of the land surface using,

(9) $$T_{B}^{p}=\frac{T_{\mathrm{hot}}-T_{\mathrm{sky}}}{U_{\mathrm{hot}}-U_{\mathrm{sky}}}U_{p}+T_{\mathrm{sky}}-\frac{T_{\mathrm{hot}}-T_{\mathrm{sky}}}{U_{\mathrm{hot}}-U_{\mathrm{sky}}}U_{\mathrm{sky}}$$

where *T*_B_ is the brightness temperature [K], *T* indicates the temperature [K] of the specified target and *U* represents the LRAD voltage observations [Volt] with subscripts *hot* and *sky* indicating the hot and *sky* target properties.

For processing the LRAD measurements to *T*_B_’s the pre-calibration was used, while the post-calibration was only employed to detect anomalous values. The estimated uncertainty of the calibrated H-polarized *T*_B_ is about ±1.0 K. While measurements were also collected for vertical polarization, there remain some unresolved issues with respect to the calibration of these measurements. Thus, V-polarized measurements are not being presented at this time.

## 4. Results

### 4.1. Surface Roughness Estimation Using H-Polarized T_B_

Within the bare soil emission model by Wang and Choudhury [25], surface roughness effects are characterized by: (1) modification of the reflectance (*h* parameter), and (2) redistribution of the H- and V-polarized emitted radiation (*Q* parameter). Since only reliably calibrated H-polarized *T*_B_ measurements are available for analysis, the *Q* parameter is omitted *i.e.,* (*Q* = 0), which essentially reduces the emission model to the one proposed by Choudhury *et al*. [36]. This formulation has been adopted previously in several other studies *i.e.,* [17,22]. Based on this assumption, the *h* parameter can be estimated from H-polarized *T*_B_’s measured over bare soil using,

(10) $$\left[1-\frac{T_{B}^{H}}{T_{s}}\right]=\left[R^{H}\;(\theta )\right]\;\text{exp}(-h)$$

where, $T_{B}^{H}$ is the H-polarized brightness temperature, T_S_ is the soil temperature, R_H_ is the H- polarized Fresnel reflectivity.

The LRAD observations during the OPE^3^ campaign started on May 22, when corn crops had just emerged and the total fresh biomass was less than 0.04 kg m^−2^. For these low biomass conditions, the measured *T*_B_’s are used to estimate the *h* parameter whereby the mean of twenty-one soil temperatures measured at a 3 cm soil depth is adopted as *T*_s_. Unfortunately, for this part of the experiment, the microwave observations were only collected from view angles of 35, 45 and 60 degrees. The *h* parameters inverted for these view angles are given in Table 1 for *G*(*θ*) functions equal to cos^2^ *θ*, cos *θ*, 1, sec *θ* and sec^2^ *θ*.

The derived *h* parameters fall within the range that has been reported previously. Wang *et al*. [26] reported a 0.00–0.53 *h* parameter range for surfaces with a *rms* height varying from 0.21 to 2.55 cm for a similar setting. Considering an averaged *rms* height of 1.62 cm was measured around the radiometer footprint, the *h* parameter values obtained from the LRAD observations appears reasonable.

A point of discussion could, however, be the angular dependence of the *h* parameter. This is absent for the 35 to 60 degrees view angle range, which is in agreement with previous reports e.g., [26,38]. An angular dependence is sometimes expected because when a radiometer observes the land surface at different angles surface roughness may have a different impact on the surface emission, while recognizing that Equation (10) is also an approximation [30]. However, the angular dependence of the *h* parameter could also be a result from the assumption *Q* = 0. The Fresnel reflectivities for the H- and V-polarization are both a function of the incidence angle; excluding one of the two polarization components, as is done by assuming *Q* = 0 in Equation (3), induces an angular dependence of the *h* parameter.

### 4.2. Surface Roughness Parameter Estimation Based on Dual-Polarized T_B_

The surface roughness parameter *h* from the present data set demonstrates an angular dependence that is equal to adopting *G*(*θ*) = 1 (see Table 1). A limitation of the present data set is that only H-polarized *T*_B_ observations are available to some degree of confidence. For retrieving the *h* parameter from these *T*_B_ values *Q* was assumed zero, which might alter the angular dependency as discussed above (mixing of polarization). To elaborate on these findings, dual polarized L-band (∼1.4 GHz) radiometer data sets collected over bare soils within the general area of the present study [23] are utilized to invert *h* and *Q* simultaneously.

The methodology used to retrieve the *Q* and *h* parameters has been adopted from Wang and Choudhury [25], which is based upon the following two relationships,

(11) $$X(\theta )=\frac{T_{\mathrm{NB}}^{V}\;(\theta )-T_{\mathrm{NB}}^{H}\;(\theta )}{1-\frac{1}{2}\left[T_{\mathrm{NB}}^{V}(\theta )+T_{\mathrm{NB}}^{H}(\theta )\right]}=2\left[\frac{R^{H}(\theta )-R^{V}\;(\theta )}{R^{H}\;(\theta )+R^{V}\;(\theta )}\right]\;(1-2Q)$$

(12) $$Y(\theta )=1-\frac{1}{2}\;\left[T_{\mathrm{NB}}^{V}(\theta )+T_{\mathrm{NB}}^{H}(\theta )\right]=\frac{1}{2}\left[R^{H}(\theta )+R^{V}\;(\theta )\right]\;\text{exp}(-\mathrm{hG}(\theta ))$$

where, $T_{\mathrm{NB}}^{p}$ is the normalized brightness temperature for polarization p, according to $T_{b}^{p}/T_{s}$, X(*θ*) is the surface roughness coefficient for deriving the *Q* parameter, Y(θ) is the surface roughness coefficient for deriving the *h* parameter.

Equations (11) and (12) can be rewritten to give the *Q* and *h* explicitly resulting in,

(13) $$\begin{array}{ccc} Q=[1-\frac{X(\theta )}{2[P(\theta )]}]/2 & \;\text{and}\; & P(\theta )=[\frac{R^{H}(\theta )-R^{V}(\theta )}{R^{H}(\theta )+R^{V}(\theta )}] \end{array}$$

with

(14) $$h=-\;\frac{\text{ln}[\frac{2Y(\theta )}{\left[R^{H}(\theta )+R^{V}(\theta )\right]}]}{G(\theta )}$$

The data set described in Wang *et al*. [26] includes ground measurements of soil moisture and temperature observed at various depths: 0–0.5, 2.5–5.0, 5.0–10.0 cm for soil moisture and 1.25, 2.5, 7.5 and 15.0 cm for soil temperature. In addition, dual-polarized *T*_B_ observations were collected at view angles of 10, 20, 30, 40, 50, 60 and 70 degrees. These measurements have been collected over soil surfaces with different roughness characteristics. For this investigation, a smooth and a rough surface are included in the analysis with a measured *rms* height of 0.73 and 2.45 cm, respectively. Because the present data set includes radiometer observations for an incidence angle range between 35 and 60 degrees, only the *T*_B_ measured over the 20 to 60 degrees incidence angle range are utilized.

**Figure 7.**
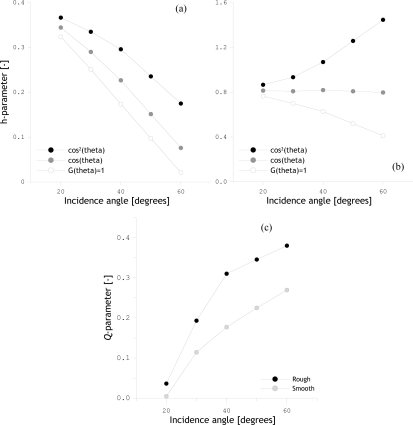
*h*-parameter as a function of incidence angle calculated from dual-polarized L-band *T*_B_’s measured over (a) smooth bare soil surface and (b) rough bare soil surface. (c) *Q*-parameters as a function of the incidence angle for same smooth and rough surfaces.

The extensiveness of the radiometer and ground measurements permits all unknowns in equations (13) and (14) to be derived, and allows the computation of surface roughness parameters *Q* and *h*. In analogy with the previous roughness computations, the soil moisture content integrated over 0–5.0 cm has been used to compute the relative dielectric constant and the soil temperature at 2.5 cm has been used to derive the normalized brightness temperature. The resulting *h* parameters are plotted as a function of the incidence angle for the rough and smooth bare soil surface in Figures 7a and 7b respectively, whereas the computed *Q* values are shown as a function of the incidence angle for both the rough and smooth surface in Figure 7c. The *h*-parameters shown in Figure 7a and 7b have been computed assuming three different *G*(*θ*) relationships, which are: *G* (*θ*) = cos^2^*θ*, cos *θ* and 1.0.

Figures 7a and 7b show a different angular behavior of the emission measured over the rough and the smooth surface. For the rough surface, it is observed that the function *G*(*θ*) = cos *θ* results in angular independent *h* parameter. However, none *G*(*θ*) functions are able to suppress the angular dependence of the *h* parameter from the smooth surface, while *G*(*θ*) = cos^2^*θ* provides the best approximation. Further, an angular dependency of *Q* parameter is noted in Figure 7c for both the rough and smooth surfaces.

The discussion above and previous results e.g., [26,36,38] indicate that consistencies in the angular dependence of roughness effect on microwave emission are difficult to identify. Hence, for SMOS soil moisture processor *h* is approximated by,

(15) $$h=h_{0}{\text{cos}}^{N_{\text{RP}}}\;\theta$$

where, *N*_RP_ quantifies the angular dependence of *h*_0_, which is also assumed to be polarization dependent.

The parameters, *h*_0_ and *N*_RH_, have been fitted to match our multi-angular data collected over nearly bare soil conditions. The obtained parameter values, and RMSE computed between the measured and retrieved *T*_B_’s and *M*_v_’s (RMSE_Tb_ and RMSD_mv_) are presented in Table 2. In addition, the optimized *h*_0_ as well as the RMSE_Tb_ and RMSD_mv_ obtained with the more frequently used *N*_RP_ values are given in Table 2.

An analysis of the parameter values shown in Table 2 demonstrates the advantage of incorporating the *N*_RP_ parameter. RMSE’s between the measured and simulated *T*_B_’s increase from about 1.2 K to more than 10.0 K when the *N*_RP_ is changed from 0 to −2 or +2. Surprisingly, this reduction in the ability to simulate *T*_B_’s only reduces the soil moisture retrieval accuracy significantly when *N*_RP_ is taken equal to −2. This is explained by the fact that the *M*_v_ is retrieved by using as cost function the RMSE computed between the *T*_B_ simulated and measured from different view angles at a given time step. For less negative and positive *N*_RP_ values, the underestimation of measured *T*_B_ at low (or high) view angles is compensated by an overestimation at high (or low) view angles. Hence, the increase in the retrieval uncertainty is for various *N*_RH_ values much smaller than would be expected based on the model‘s ability to simulate T_B_’s. It should, however, be noted that in this case only bare soil conditions are considered and, thus, results may be different under vegetation conditions.

### 4.3. Estimation of the H-polarized Transmissivity

When soil moisture and surface temperature are known, H-polarized transmissivity (*γ*_h_) can be estimated through the inversion of Equation (1) assuming that temporal variations in the roughness parameters are small and the *ω* equal to zero. Estimates of the *γ*_h_ are only presented for retrievals from H-polarized *T*_B_’s measured in the early morning (around 8:00 AM) because at that time of the day the soil surface and vegetation are typically found to be in thermal equilibrium e.g., [40]. This assumption permits using a single so-called ‘effective’ temperature as input for Equation (1), for which the temperature measured at a 3 cm soil depth is adopted. Further, for calculation of the Fresnel reflectivity, the *ε*_r_ is obtained through application of Dobson‘s soil mixing model [35] with input of soil textural properties and the measured soil moisture.

**Figure 8.**
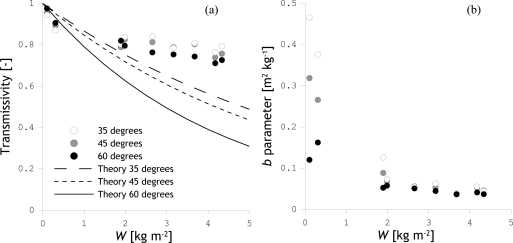
H-polarized corn transmissivities (a) and b parameters (b) inverted from *T*_B_’s measured at incidence angles of 35, 45 and 60 degrees plotted against the total plant *W*.

The retrieved *γ*_h_ for each day and view angles of 35, 45 and 60 degrees are given in Table 3 and are plotted in Figure 8a against the total plant *W*. In Figure 8a, *γ*_h_ computations are also presented for an assumed *b* parameter value of 0.117 m^2^ kg^−1^, which is the median of L-band corn *b* values presented in Jackson and Schmugge [20]. Further, *b* parameters have been derived from the retrieved *γ*_h_’s, which are given in Table 3 and plotted against the total plant *W* in Figure 8b. It should be noted that most *b* parameters have previously been derived for dense corn canopies with *W* in the range 1.2−6.0 kg m^−2^. A comparison of *b* parameters derived for May 29 and June 5 (*W* = 0.1 and 0.3 kg m^−2^) against previously reported values is, therefore, not optimal. The field conditions observed from June 19 to August 30 (*W* = 1.9 – 4.3 kg m^−2^) are, however, compatible in terms of biomass to corn canopies referred to in these previous investigations.

Figures 8a shows that the retrieved *γ*_h_ follows a different pattern than expected based on the literature. At the beginning of the growth cycle, the *γ*_h_ is smaller than expected, while closer to peak biomass the *γ*_h_ is larger. In terms of the *b* parameter, the obtained values are higher than the literature reports just after emergence of the corn crops and somewhat lower at higher *W* levels (>1.9 kg m^−2^). The dependence of the *b* parameter on *W* can be argued based on previous investigations. Le Vine and Karam [41], among others, have shown that the attenuation by canopies composed of elements with similar dimensions as the wavelength is also specific to the vegetation morphology. As changes in biomass (or *W*) are typically associated with different growth stages and also architectural changes in the canopy, the *b* parameter can be expected to vary throughout the growth cycle.

On the other hand, it should be noted that the presented *γ*_h_’s and *b* parameters are also subject to various sources of uncertainty embedded within the inversion procedure. For example, corn crops at the beginning of the growing season are very small, which lead to relatively large uncertainties in the measured *W.* Moreover, the contribution of the vegetation emission to the measured *T*_B_ is also small at the early growth stage. Uncertainties in the measured *T*_B_ may have, therefore, a large impact on the derived *b* parameters. To demonstrate the impact of such *T*_B_ uncertainties on the derivation of *b* parameters from measurements acquired over sparse and dense vegetation, the *γ*_h_’s have been inverted after perturbing the *T*_B_ measured on May 29th (*W* = 0.1 kg m^−2^) and July 9th (*W* = 4.2 kg m^−2^) by ±1.0 K. The obtained *γ*_h_’s and *b* parameters for these two dates are given in Table 4. These results confirm that under sparsely vegetated conditions *T*_B_ uncertainties have a larger impact on the derived *b* parameters than under densely vegetation conditions. The *b* values retrieved for May 29th range from 0.355 to 0.510 m^2^ kg^−1^ for the 35 degrees view angle, while for the same angle the *b* parameter from July 9th range from 0.073 to 0.106 m^2^ kg^−1^.

The somewhat higher *γ*_h_’s (and lower *b* parameters) obtained over more dense vegetation are explained by the effects of scattering within the canopy, which has not been considered as *ω* = 0.0 has been assumed. When the attenuation by vegetation is small, the scattering within the canopy can be assumed negligible because the emission by vegetation is small e.g., [14]. This justifies using *ω* = 0.0. As the biomass increases, vegetation emission also increases and scattering within the canopy will have a more important impact on the measured *T*_B_. The previously reported *ω* values tabulated in Van de Griend and Wigneron [37] may reach for L-band and corn up to values of 0.13.

To quantify this effect of scattering under densely vegetated conditions, the *ω* is inverted, instead of *γ*_h_, from the *T*_B_’s measured on July 9th for assumed *b* parameters of 0.10, 0.11, 0.12, 0.13, 0.14 and 0.15 m^2^ kg^−1^. The obtained *ω*’s are given in Table 5, which illustrate the numerical correlation between the parameters *b* and *ω* within *T*_B_ simulations using Equation (1); namely for small *b* parameters, *ω* is also small. Further, it is noted that the inverted *ω*’s are dependent on the view angle. This can be argued for since scattering within the canopy is affected by orientation of scatterers (e.g., stems, leaves and cobs) relative to the view angle e.g., [27,28].

The previous discussion on the effects of vegetation on L-band H-polarized *T*_B_’s demonstrates that the strength of scattering and absorption within a corn canopy changes over the growth cycle. This can be attributed to changes in the canopy‘s architecture as the corn crops develop. As a result, the *τ*_h_ is found to be a nonlinear function of the *W*, while most of current soil moisture retrieval algorithms adopt a linear relationships. To evaluate how this assumption influences the reliability of retrievals, soil moisture is inverted by minimizing RMSE between simulated and measured *T*_B_’s for view angles of 35, 45, and 60 degrees and assuming a constant *b* and *ω* of 0.12 m^2^ kg^−1^ and 0.0 based on [30], respectively.

**Figure 9.**
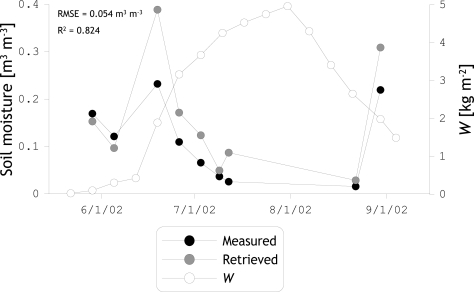
Soil moisture measurements and retrievals obtained by assuming a constant *b* parameter and *ω* of 0.12 m^2^ kg^−1^ and 0.0, respectively.

The retrieved and measured soil moisture is plotted against time along with the total plant *W* in Figure 9. The plot shows an underestimation of measured soil moisture over sparse vegetation (*W* < 1.0 kg m^−2^) and an overestimation for densely vegetated conditions (*W* > 1.5 kg m^−2^). As the contribution of vegetation on both *T*_B_ measurements and simulations is more significant at a high biomass, the imperfect vegetation parameterization leads to a larger overestimation for dense vegetation as compared to the underestimation found for sparse vegetation; RMSE = 0.021 m^3^ m^−3^ for *W* < 1.0 kg m^−2^ and RMSE = 0.065 m^3^ m^−3^ for *W* > 1.0 kg m^−2^. Based on these results it may be concluded that consideration of the canopy’s architecture for determining the vegetation parameters will assist in further improving the reliability of soil moisture retrievals especially over dense vegetation.

## 5. Concluding Remarks

In this investigation, the H-polarized *T*_B_’s measured by a tower mounted L-band (1.4 GHz) radiometer were used to analyze the vegetation effects on surface emission throughout the 2002 corn growth cycle. Concurrent with the radiometer measurements an extensive land surface characterization took place about once a week including soil moisture, soil temperature and vegetation biomass measurements. Over the period from May 22 to August 30, ten days with a complete record of ground and radiometer measurements are available for analysis covering a vegetation water content (*W*) range of 0.0 to 4.3 kg m^−2^.

The roughness parameter, *h*, needed to correct for the effects of surface roughness, was inverted from H-polarized *T*_B_ measured early in the corn growing season over essentially a bare soil surface. Since V-polarized *T*_B_ measurements were not available for this investigation, the surface emission model by Choudhury *et al*. [34] (assuming *Q* = 0.0) was adopted and different *G* (*θ*) functions were used to analyze the angular dependence of *h*. While recognizing that both V- and H-polarized reflectivities depend on the view angle, the assumption *Q* = 0.0 could affected the obtained angular dependency of *h*. Therefore, a dual-polarized L-band radiometer data set from 1981 [26] was used to investigate the impact of assuming *Q* equal to 0.0. It was found that even within this complete radiometer data sets consistencies in the angular dependence of the *h* are difficult to identify, which is in line with the parameterization *G*(*θ*) = cos^NRP^(*θ*) adopted for the SMOS level 2 soil moisture processor. Using this formulation, a good agreement was obtained between the measured and computed *T*_B_.

Based on the derived surface roughness formulation, H-polarized corn transmissivities (*γ*_h_) have been retrieved using the radiative transfer equation and assuming the single scattering albedo (*ω*_h_) equal to zero. The derived *γ*_h_’s were converted into *b* parameter values using the measured total plant *W*. For sparse vegetation, the obtained *τ*_h_’s and *b* parameters were found to be larger than the values reported in the literature. This is partly explained by the fact that under low biomass conditions *T*_B_ uncertainties result in a particularly large uncertainty in the derived *b* parameter. For dense vegetation, the inverted *b* parameters were somewhat smaller than expected, which was attributed to scattering within the canopy that was not accounted for, since *ω* was initially assumed to be zero. By assuming that the corn *b* parameter varies from 0.10 to 0.15 m^2^ kg^−1^, the *ω*_h_ was derived from *T*_B_ measurements. For this range of *b* parameters, the obtained range in *ω*_h_’s is in agreement with literature reports, but displays a strong angular dependence.

This study shows that the strength of scattering and absorption within a corn canopy changes throughout the growth cycle, which can be largely attributed to changes in architecture of vegetation layer. For further improvement of the soil moisture retrieval reliability over dense vegetated conditions the canopy’s architecture should be taken into consideration for determining vegetation parameters. Analysis of additional radiometer data sets and simulations by advanced vegetation scattering models is recommended to obtain a more thorough understanding of the behavior of the *b* parameters throughout the growth cycle.

## Figures and Tables

**Table 1.** Surface parameters obtained through inversion of H-polarized TB observations acquired over bare soil conditions.

	***View angle***		
	35 degrees	45 degrees	60 degrees

*h = h_0_·cos^2^ θ*	0.641	0.867	1.663
*h = h_0_·cos θ*	0.525	0.613	0.832
*h = h_0_*	0.429	0.434	0.416
*h = h_0_·sec θ*	0.352	0.307	0.208
*h = h_0_·sec^2^ θ*	0.288	0.217	0.104

**Table 2.** *h*_0_ parameter inverted using multi angular H-polarized *T*_B_’s measured over bare soil and assuming different *N*_RH_ values, in bold are the *h*_0_ and *N*_RH_ parameters simultaneously inverted from the multi angular data.

		***RMSE***	

***N*_RH_**	***h*_0_**	***T*_B_ [K]**	***M*_v_ [m^3^ m^−3^]**
*N*_RH_ = 0.05 (opt.)^*^	**0.411**	**1.205**	**0.0053**
*N*_RH_ = −2	0.104	11.225	0.0616
*N*_RH_ = −1	0.277	6.155	0.0074
*N*_RH_ = 0	0.407	1.208	0.0065
*N*_RH_ = 1	0.613	7.441	0.0082
*N*_RH_ = 2	0.784	13.734	0.0074

* *N*_RH_ and *h*_0_ are calibrated simultaneously.

**Table 3.** H-polarized transmissivities and *b* parameters estimated over the 2002 corn growth cycle using multi angular brightness temperatures.

**Date**	***W***	***Transmissivity***			***B parameter***		
	*kg m^−2^*	*35°*	*45°*	*60°*	*35°*	*45°*	*60°*

*May 29, 2002*	0.1	0.945	0.951	0.967	0.431	0.356	0.167
*June 5, 2002*	0.3	0.857	0.878	0.881	0.423	0.306	0.211
*June 19, 2002*	1.9	0.784	0.830	0.763	0.105	0.070	0.071
*June 26, 2002*	3.1	0.678	0.684	0.641	0.101	0.085	0.070
*July 3, 2002*	3.7	0.695	0.679	0.629	0.081	0.075	0.063
*July 9, 2002*	4.2	0.640	0.552	0.556	0.088	0.101	0.070
*July 12, 2002*	4.3	0.639	0.532	0.517	0.085	0.103	0.076
*August 21, 2002*	2.6	0.783	0.758	0.736	0.078	0.076	0.060
*August 30, 2002*	2.0	0.821	0.786	0.732	0.081	0.086	0.079

**Table 4.** H-polarized transmissivities and *b* parameters inverted from *T*_B_’s measured under sparsely (May 29th, *W* = 0.1 kg m^−2^) and densely (July 9th, *W* = 4.2 kg m^−2^) vegetated conditions and perturbed by +1.0, 0.0 and −1.0 K, respectively.

		***Transmissivity***			***b-parameter***		
		*35°*	*45°*	*60°*	*35°*	*45°*	*60°*

May 29th *W* = 0.1 kg m^−2^	*TB − 1.0 K*	0.958	0.958	0.973	0.355	0.300	0.139
	*TB*	0.949	0.951	0.967	0.431	0.356	0.167
	*TB + 1.0 K*	0.940	0.943	0.962	0.510	0.413	0.195

July 9th *W* = 4.2 kg m^−2^	*TB − 1.0 K*	0.690	0.593	0.577	0.073	0.089	0.066
	*TB*	0.640	0.552	0.556	0.088	0.101	0.070
	*TB + 1.0 K*	0.584	0.506	0.535	0.106	0.116	0.075

**Table 5.** Single scattering albedo (*ω*) inverted from LRAD *T*_B_ measured on June 9, 2002 (*W =* 4.2 kg m^−2^) assuming a range *b* parameters from 0.10 to 0.15 m^2^ kg^−1^.

**b-parameter**	***Single albedo***	***scattering***	
*m^2^ kg^−1^*	*35°*	*45°*	*60°*

0.10	0.014	0.017	0.033
0.11	0.016	0.021	0.037
0.12	0.018	0.024	0.040
0.13	0.020	0.027	0.043
0.14	0.021	0.028	0.044
0.15	0.022	0.030	0.045
